# A Novel Approach toward Fuzzy Generalized Bi-Ideals in Ordered Semigroups

**DOI:** 10.1155/2014/275947

**Published:** 2014-04-27

**Authors:** Faiz Muhammad Khan, Nor Haniza Sarmin, Hidayat Ullah Khan

**Affiliations:** ^1^Department of Mathematics and Statistics, University of Swat, Swat, Khyber Pakhtunkhwa 19130, Pakistan; ^2^Department of Mathematical Sciences, Faculty of Science, Universiti Teknologi Malaysia, UTM, 81310 Johor Bahru, Johor, Malaysia

## Abstract

In several advanced fields like control engineering, computer science, fuzzy automata, finite state machine, and error correcting codes, the use of fuzzified algebraic structures especially ordered semigroups plays a central role. In this paper, we introduced a new and advanced generalization of fuzzy generalized bi-ideals of ordered semigroups. These new concepts are supported by suitable examples. These new notions are the generalizations of ordinary fuzzy generalized bi-ideals of ordered semigroups. Several fundamental theorems of ordered semigroups are investigated by the properties of these newly defined fuzzy generalized bi-ideals. Further, using level sets, ordinary fuzzy generalized bi-ideals are linked with these newly defined ideals which is the most significant part of this paper.

## 1. Introduction

The major advancements in the fascinating world of fuzzy set started in 1965 with new directions and ideas. A fuzzy set can be defined as a set without a crisp and clearly sharp boundaries which contains the elements with only a partial degree of membership. Fuzzy sets are the extensions of classical sets. The quest for the fuzzification of algebraic structures was long considered an unreasonable target, until Rosenfeld's fuzzy subgroups concept [1]. The latest advances in the investigation of fuzzy subgroup theory have drawn increasing interest to this class of algebraic structures. This knowledge of Rosenfeld's concept is also of fundamental importance in the most important generalization, that is, (∈, ∈∨*q*)-fuzzy subgroups. The concept that belongs to relation (∈) and quasicoincident with relation (*q*) relation of a fuzzy point to fuzzy set was introduced by Pu and Liu [2] and has increased the importance of algebraic structures. A fuzzy point [*x*; *t*] belongs to (resp., quasicoincident with) a fuzzy set *μ*, if *μ*(*x*) ≥ *t* (resp., *μ*(*x*) + *t* > 1) and is denoted by [*x*; *t*] ∈ *μ* (resp., [*x*; *t*]*qμ*), where *t* ∈ (0,1]. The idea of a quasi-coincidence of a fuzzy point with fuzzy set played a significant role in generating different types of fuzzy subgroups. Bhakat and Das [3] used the notions of “belongs to relation” and “quasicoincident with relation” and proposed the idea of fuzzy subgroups of type (*α*, *β*), where *α*, *β* ∈ {∈, *q*, ∈∨*q*, ∈∧*q*} and *α* ≠ ∈∧*q*. The idea of generalized fuzzy subgroups has increased the importance of algebraic structures by attracting the attention of many researchers and opened ways for future researchers in this field. Furthermore, Jun [4] generalized the concept of “quasicoincident with relation” and defined a new relation (*q*_*k*_), where *k* ∈ [0,1).

The idea of belongs to relation (∈) and quasicoincident with relation (*q*) relation is further applied in semigroups to investigate some new types of interior ideals. Therefore, the concept of a (*α*, *β*)-fuzzy interior ideal in semigroups is introduced by Jun and Song [5]. Furthermore, this concept is extended to ordered semigroups where Khan and Shabir [6] initiate (*α*, *β*)-fuzzy interior ideals in ordered semigroups and discussed some basic properties of (*α*, *β*)-fuzzy interior ideals. In semigroup, Kazanci and Yamak [7] introduced fuzzy bi-ideal with thresholds, (∈, ∈∨*q*)-fuzzy bi-ideals, and ($\bar{\in },\bar{\in }\vee \bar{q}$)-fuzzy bi-ideals, which are generalizations of the concept of fuzzy bi-ideals, whereas in ordered semigroups, Jun et al. [8] gave the idea of (∈, ∈∨*q*)-fuzzy bi-ideals, which is a generalization of the concept of a fuzzy bi-ideal in ordered semigroups. Davvaz and Khan [9] discussed some characterizations of regular ordered semigroups in terms of (*α*, *β*)-fuzzy generalized bi-ideals, where *α*, *β* ∈ {∈, *q*, ∈∨*q*, ∈∧*q*} and *α* ≠ ∈∧*q*. By using the idea given in [4] Shabir et al. [10] gave the concept of more general form of (*α*, *β*)-fuzzy ideals and initiated (∈, ∈∨*q*_*k*_)-fuzzy ideals of semigroups, where *k* ∈ [0,1).

In 2010, Yin and Zhan [11] introduced more general forms of (∈, ∈∨*q*)-fuzzy (implicative, positive implicative, and fantastic) filters and ($\bar{\in },\bar{\in }\vee \bar{q}$)-fuzzy (implicative, positive implicative, and fantastic) filters of *BL*-algebras, and defined (∈_*γ*_, ∈_*γ*_∨*q*_*δ*_)-fuzzy (implicative, positive implicative and fantastic) filters and ($\bar{\in_{\gamma }},\bar{\in_{\gamma }}\vee \bar{q_{\delta }}$)-fuzzy (implicative, positive implicative and fantastic) filters of *BL*-algebras and gave some interesting results in terms of these notions. The importance of these new types of notion is further increased by the reports of Ma et al. [12] who introduced the concept of (∈_*γ*_, ∈_*γ*_∨*q*_*δ*_)-fuzzy ideals and ($\bar{\in_{\gamma }},\bar{\in_{\gamma }}\vee \bar{q_{\delta }}$)-fuzzy ideals of *BCI*-algebras and discussed several important results of *BCI*-algebras in terms of these new types of notions. Further, Khan et al. [13] initiated (∈_*γ*_, ∈_*γ*_∨*q*_*δ*_)-fuzzy interior ideals in ordered semigroups and characterized ordered semigroups by the properties of these new types of fuzzy interior ideals. These innovative types of fuzzy ideals are also investigated by Khan et al. [14] in *AG*-groupoids and funded out several important results of *AG*-groupoids on the basis of (∈_*γ*_, ∈_*γ*_∨*q*_*δ*_)-fuzzy ideals.

Inspired by these outstanding findings, based on Yin and Zhan [11] and Ma et al. [12] idea, we introduce a more generalized form of (∈, ∈∨*q*)-fuzzy generalized bi-ideals called (*α*, *β*)-fuzzy generalized bi-ideals of an ordered semigroup *S*, where *α*, *β* ∈ {∈_*γ*_, *q*_*δ*_, ∈_*γ*_∧*q*_*δ*_, ∈_*γ*_∨*q*_*δ*_} with *α* ≠ ∈_*γ*_∧*q*_*δ*_ and discuss several important and fundamental aspects of ordered semigroups in terms of (∈_*γ*_, ∈_*γ*_∨*q*_*δ*_)-fuzzy generalized bi-ideals and ($\bar{\in_{\gamma }},\bar{\in_{\gamma }}\vee \bar{q_{\delta }}$)-fuzzy generalized bi-ideals. Several examples are constructed to support these new types of fuzzy generalized bi-ideals. Since it is known that every bi-ideal is generalized bi-ideal but the converse is not true, therefore, in these new types of fuzzy generalized bi-ideals every (∈_*γ*_, ∈_*γ*_∨*q*_*δ*_)-fuzzy bi-ideal is an (∈_*γ*_, ∈_*γ*_∨*q*_*δ*_)-fuzzy generalized bi-ideal. An example is constructed which shows that the converse of the aforementioned statement is not true in general. We also defined ($\bar{\beta },\bar{\alpha }$)-fuzzy generalized bi-ideals and some related properties are investigated, where $\bar{\alpha },\bar{\beta }\in \{\bar{\in_{\gamma }},\bar{q_{\delta }},\bar{\in_{\gamma }}\wedge \bar{q_{\delta }},\bar{\in_{\gamma }}\vee \bar{q_{\delta }}\}$ and $\bar{\beta }\neq \bar{\in_{\gamma }}\wedge \bar{q_{\delta }}$. The innovativeness of this paper is to establish relationships among ordinary fuzzy generalized bi-ideals, (∈_*γ*_, ∈_*γ*_∨*q*_*δ*_)-fuzzy generalized bi-ideals, and ($\bar{\in_{\gamma }},\bar{\in_{\gamma }}\vee \bar{q_{\delta }}$)-fuzzy generalized bi-ideals by using level subsets.

## 2. Preliminaries

An* ordered semigroup* is a structure (*S*, ·, ≤) in which (*S*, ·) is a semigroup and (*S*, ≤) is a poset and if *a* ≤ *b*, then *ax* ≤ *bx* and *xa* ≤ *xb* for all *a*, *b*, *x* ∈ *S*. Note that throughout the paper *S* is an ordered semigroup unless otherwise stated. For *A*, *B*⊆*S* we denote (*A*] = {*t* ∈ *S* | *t* ≤ *h* for some *h* ∈ *A*}, and *AB* = {*ab* ∈ *S* | *a* ∈ *A*, *b* ∈ *B*}. If *A* = {*a*}, then we write (*a*] instead of ({*a*}]. If *A*, *B*⊆*S*, then *A*⊆(*A*], (*A*](*B*]⊆(*AB*], and ((*A*]] = (*A*].

Let *S* be an ordered semigroup. A nonempty subset *A* of *S* is called a* subsemigroup* of *S* if *A* ^2^⊆*A*.

**Definition 1 (see [15])**

A nonempty subset *A* of an ordered semigroup *S* is called a* generalized bi-ideal* of *S* if

- (∀*a* ∈ *S*) (∀*b* ∈ *A*) (*a* ≤ *b* → *a* ∈ *A*),
- *ASA*⊆*A*.

**Definition 2 (see [16])**

A nonempty subset *A* of an ordered semigroup *S* is called a* bi-ideal* of *S* if

- (∀*a* ∈ *S*) (∀*b* ∈ *A*) (*a* ≤ *b* → *a* ∈ *A*),
- *ASA*⊆*A*,
- *A* ^2^⊆*A*.

By Definitions 1 and 2 it is clear that every bi-ideal is a generalized bi-ideal, but the converse is not true.

**Example 3 (see [17])**

Consider the ordered semigroup *S* = {0, *x*, *y*, *z*} with ordered relations {(0,0), (*x*, *x*), (*y*, *y*), (*z*, *z*), (0, *x*)} and the following multiplication table:

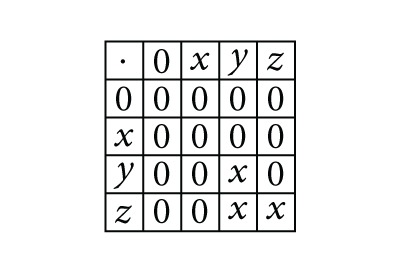
(1)

The bi-ideals of *S* are {0}, {0, *x*}, {0, *x*, *y*}, {0, *x*, *z*} and {0, *x*, *y*, *z*}, where the generalized bi-ideals of *S* are {0}, {0, *x*}, {0, *y*}, {0, *z*}, {0, *x*, *y*}, {0, *x*, *z*}, {0, *y*, *z*}, and {0, *x*, *y*, *z*}. One can check that {0, *y*}, {0, *z*}, and {0, *y*, *z*} are not bi-ideals.

It is important to note that several mathematical phenomena being vague and probabilistic in nature cannot be manipulated by classical sets. Zadeh [18] was the icebreaker to pioneer the idea of fuzzy subset (extension of classical sets) of a set, which could address these kinds of problems.

Now, we give some fuzzy logic concepts.

**Definition 4 (see [18])**

A* fuzzy subset μ* from a universe *X* is a function from *X* into a unit closed interval [0,1] of real numbers.

After the introduction of fuzzy set theory [18], Rosenfeld [1] initiated the fuzzification of algebraic structures and introduced the notion of fuzzy groups and successfully extended many results from groups to the theory of fuzzy groups. In semigroups the theory of fuzzy ideals, fuzzy bi-ideals, and fuzzy quasi-ideals is given by Kuroki [19–22].

A fuzzy subset *μ* of *S* is called a* fuzzy subsemigroup* if for all *x*, *y* ∈ *S*,

(2) $$\begin{array}{c} \mu \left(xy\right)\geq \min\left\{\mu \left(x\right),\mu \left(y\right)\right\}. \end{array}$$

**Definition 5 (see [17])**

A fuzzy subset *μ* of *S* is called a* fuzzy generalized bi-ideal* of *S* if for all *x*, *y*, *z* ∈ *S* the following conditions hold:

- *x* ≤ *y* → *μ*(*x*) ≥ *μ*(*y*),
- *μ*(*xyz*) ≥ min⁡{*μ*(*x*), *μ*(*z*)}.

**Definition 6 (see [16])**

A fuzzy subsemigroup *λ* is called a* fuzzy bi-ideal* of *S* if the following conditions hold for all *x*, *y*, *z* ∈ *S*:

- *x* ≤ *y* → *μ*(*x*) ≥ *μ*(*y*),
- *μ*(*xyz*) ≥ min⁡{*μ*(*x*), *μ*(*z*)}.

Note that every fuzzy bi-ideal is a generalized fuzzy bi-ideal of *S*. But the converse is not true, as given in [14].

If *μ* is a fuzzy subset of *S*, then the set *U*(*μ*; *t*) = {*x* ∈ *S* | *μ*(*x*) ≥ *t*} is called a* level set* of *μ* for all *t* ∈ (0,1].

**Theorem 7 (see [9])**

A fuzzy subset *μ* of an ordered semigroup *S* is a fuzzy generalized bi-ideal of *S* if and only if *U*(*μ*; *t*)(≠*∅*), where *t* ∈ (0,1] is a generalized bi-ideal of *S*.

**Theorem 8 (see [9])**

A nonempty subset *A* of an ordered semigroup *S* is a generalized bi-ideal of *S* if and only if

(3) $$\begin{array}{c} \chi_{A}:S⟶\left[0,1\right]\;|\;x⟼\chi_{A}\left(x\right)=\{\begin{array}{cc} 1 & if\;x\in A,\\ 0 & if\;x\notin A, \end{array} \end{array}$$

is a fuzzy generalized bi-ideal of *S*.

**Definition 9 (see [2])**

Let *μ* be a fuzzy subset of *S*; then the set of the form:

(4) $$\begin{array}{c} \mu \left(y\right):=\{\begin{array}{ccc} t, & \text{if}\;\;y=x, & \\ 0, & \text{if}\;\;y\neq x, & \end{array} \end{array}$$

is called a* fuzzy point* with support *x* and value *t* and is denoted by [*x*; *t*]. A fuzzy point [*x*; *t*] is said to* belong to* (resp., quasicoincident with) a fuzzy set *λ*, written as [*x*; *t*] ∈ *λ* (resp., [*x*; *t*]*qλ*) if *λ*(*x*) ≥ *t* (resp., *λ*(*x*) + *t* > 1). If [*x*; *t*] ∈ *λ* or [*x*; *t*]*qλ*, then we write [*x*; *t*]∈∨*qλ*. The symbol $\bar{\in \vee q}$ means that ∈∨*q* does not hold.

**Definition 10 (see [11])**

A fuzzy subset *μ* of *S* is called an (∈, ∈∨*q*)*-fuzzy generalized bi-ideal* of *S* if it satisfies the following conditions:

- (∀*x*, *a*, *y* ∈ *S*) (∀*t*, *r* ∈ (0,1])  ([*x*; *t*] ∈ *μ*[*y*; *r*], ∈*μ*⇒[*xa* *y*; min⁡{*t*, *r*}]∈∨*qμ*),
- (∀*x*, *y* ∈ *S*) (∀*t* ∈ (0,1])  (*x* ≤ *y*[*y*; *t*], ∈*μ*⇒[*x*; *t*]∈∨*qμ*).

**Theorem 11 (see [11])**

A fuzzy subset *μ* of *S* is an (∈, ∈∨*q*)-fuzzy generalized bi-ideal of *S* if and only if it satisfies the following conditions:

- (∀*x*, *a*, *y* ∈ *S*)  (*μ*(*xa* *y*) ≥ min⁡{*μ*(*x*), *μ*(*y*), 0.5}),
- (∀*x*, *y* ∈ *S*)  (*x* ≤ *y*, *μ*(*x*) ≥ min⁡{*μ*(*y*), 0.5}).

**Definition 12**

A fuzzy subset *μ* of *S* is called an $\left(\bar{\in },\bar{\in }\vee \bar{q}\right)$ *-fuzzy generalized bi-ideal* of *S* if it satisfies the following conditions:

1. (∀*x*, *y* ∈ *S*)  (∀*r* ∈ (0,1])$([x;r]\bar{\in }\mu \Rightarrow [y;r]\bar{\in }\vee \bar{q}\mu$ with *x* ≤ *y*),
2. (∀*x*, *a*, *y* ∈ *S*)  (∀*r*, *t* ∈ (0,1])$([xay;\min\{r,t\}]\bar{\in }\mu \Rightarrow [x;r]\bar{\in }\vee \bar{q}\mu$ or $\left[y;t]\bar{\in }\vee \bar{q}\mu \right)$.

**Example 13**

Consider *S* = {*a*, *b*, *c*, *d*, *e*} with the following multiplication table and order relation:

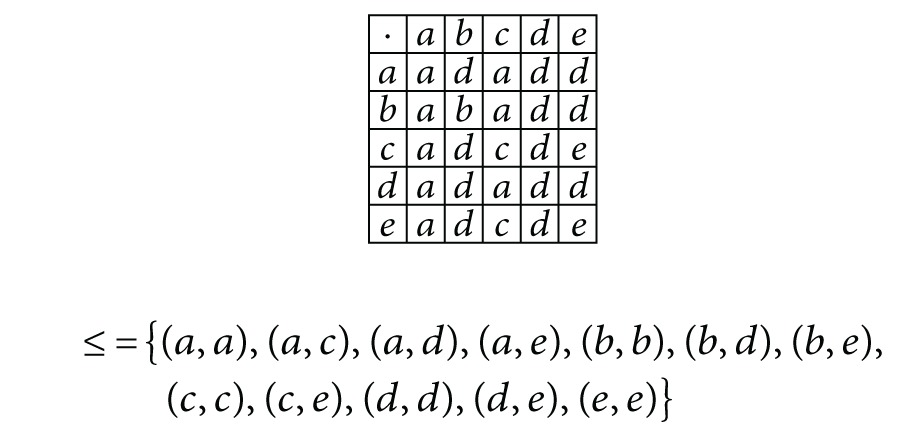
(5)

Define a fuzzy subset *μ* : *S* → [0,1] as follows:

(6) $$\begin{array}{c} \mu \left(x\right)=\{\begin{array}{cc} 0.80, & \text{if}\;x=a,\\ 0.50, & \text{if}\;x=b,c,d,e. \end{array} \end{array}$$

Then by Definition 12 *μ* is an ($\bar{\in },\bar{\in }\vee \bar{q}$)-fuzzy generalized bi-ideal of *S*.

**Theorem 14**

A fuzzy subset *μ* of *S* is an ($\bar{\in },\bar{\in }\vee \bar{q}$)-fuzzy generalized bi-ideal of *S* if and only if

(3) (∀*x*, *y* ∈ *S*)  (max⁡{*μ*(*x*), 0.5} ≥ *μ*(*y*) with *x* ≤ *y*),
(4) (∀*x*, *a*, *y* ∈ *S*)  (max⁡{*μ*(*xa* *y*), 0.5} ≥ min⁡{*μ*(*x*), *μ*(*y*)}).

**Proof**

(1)⇒(3). If there exists *x*, *y* ∈ *S* with *x* ≤ *y* such that

(7) $$\begin{array}{c} \max\left\{\mu \left(x\right),0.5\right\}<r=\mu \left(y\right), \end{array}$$

then 0.5 < *t* ≤ 1, $\left[x;r\right]\bar{\in }\mu$ but [*y*; *t*] ∈ *μ*. By (1), we have $[y;r]\bar{q}\mu$. Then *μ*(*y*) ≥ *t* and *r* + *μ*(*y*) ≤ 1, which implies that *t* ≤ 0.5, a contradiction. Hence (3) is valid.

(3)⇒(1). Let *x*, *y* ∈ *S* with *x* ≤ *y* and *r* ∈ (0,1] be such that $[x;r]\bar{\in }\mu$. Then *μ*(*x*) < *r*. If *μ*(*x*) ≥ *μ*(*y*), then *μ*(*y*) ≤ *μ*(*x*) < *r*. It follows that $[y;r]\bar{\in }\mu$. If *μ*(*x*) < *μ*(*y*), then by (3), we have 0.5 ≥ *μ*(*y*). Let $[y;r]\bar{\in }\mu$, then *μ*(*y*) < *r* and *μ*(*y*) ≤ 0.5. It follows that $\left[y;r\right]\bar{q}\mu$; thus $[y;r]\bar{\in }\vee \bar{q}\mu$.

(2)⇒(4). If there exists *x*, *a*, *y* ∈ *S* such that

(8) $$\begin{array}{c} \max\left\{\mu \left(xay\right),0.5\right\}<t=\min\left\{\mu \left(x\right),\mu \left(y\right)\right\}, \end{array}$$

then 0.5 < *t* ≤ 1, $\left[xay;t\right]\bar{\in }\mu$ but [*x*; *t*] ∈ *μ* and [*y*; *t*] ∈ *μ*. By (2), we have $[x;t]\bar{q}\mu$ or $[y;t]\bar{q}\mu$. Then (*μ*(*x*) ≥ *t* and *t* + *μ*(*x*) ≤ 1) or (*μ*(*y*) ≥ *t* and *t* + *μ*(*y*) ≤ 1), which implies that *t* ≤ 0.5, a contradiction.

(4)⇒(2). Let *x*, *a*, *y* ∈ *S* and *r*, *t* ∈ (0,1] be such that $[xay;\min\{r,t\}]\bar{\in }\mu$; then *μ*(*xa* *y*) < min⁡{*r*, *t*}.

(a) If *μ*(*xa* *y*) ≥ min⁡{*μ*(*x*), *μ*(*y*)}, then min⁡{*μ*(*x*), *μ*(*y*)} < min⁡{*r*, *t*} and consequently *μ*(*x*) < *r* or *μ*(*y*) < *t*. It follows that $[x;r]\bar{\in }\mu$ or $[y;t]\bar{\in }\mu$. Thus $[x;r]\bar{\in }\vee \bar{q}\mu$ or $[y;t]\bar{\in }\vee \bar{q}\mu$.

(b) If *μ*(*xa* *y*) < min⁡{*μ*(*x*), *μ*(*y*)}, then by (4), we have 0.5 ≥ min⁡{*μ*(*x*), *μ*(*y*)}. Let $[x;r]\bar{\in }\mu$ or $\left[y;t\right]\bar{\in }\mu$; then *μ*(*x*) < *r* and *μ*(*x*) ≤ 0.5 or *μ*(*y*) < *t* and *μ*(*y*) ≤ 0.5. It follows $[x;r]\bar{q}\mu$ or $[y;t]\bar{q}\mu$ and $[x;r]\bar{\in }\vee \bar{q}\mu$ or $[y;t]\bar{\in }\vee \bar{q}\mu$.

## 3. (*α*, *β*)-Fuzzy Generalized Bi-Ideals

From the time that fuzzy subgroups gained general acceptance over the decades, it has provided a central trunk to investigate similar type of generalization of the existing fuzzy subsystems of other algebraic structures. A contributing factor for the growth of fuzzy subgroups is increased by the reports from Yin and Zhan [11] and Ma et al. [12] who introduced the concept of (∈_*γ*_, ∈_*γ*_∨*q*_*δ*_)-fuzzy filters, ($\bar{\in_{\gamma }},\bar{\in_{\gamma }}\vee \bar{q_{\delta }}$)-fuzzy filters of *BL*-algebras and (∈_*γ*_, ∈_*γ*_∨*q*_*δ*_)-fuzzy ideals, and ($\bar{\in_{\gamma }},\bar{\in_{\gamma }}\vee \bar{q_{\delta }}$)-fuzzy ideals of *BCI*-algebras, respectively. In this section, we introduce some new types of relationships between fuzzy points and fuzzy subsets and investigate (*α*, *β*)-fuzzy generalized bi-ideals of ordered semigroups.

In what follows let *γ*, *δ* ∈ [0,1] be such that *γ* < *δ*. For a fuzzy point [*x*; *t*] and a fuzzy subset *μ* of *X*, we say that

1. [*x*; *t*]∈_*γ*_ *μ* if *μ*(*x*) ≥ *t* > *γ*.
2. [*x*; *t*]*q*_*δ*_ *μ* if *μ*(*x*) + *t* > 2*δ*.
3. [*x*; *t*]∈_*γ*_∨*q*_*δ*_ *μ* if [*x*; *t*]∈_*γ*_ *μ* or [*x*; *t*]*q*_*δ*_ *μ*.
4. [*x*; *t*]∈_*γ*_∧*q*_*δ*_ *μ* if [*x*; *t*]∈_*γ*_ *μ* and [*x*; *t*]*q*_*δ*_ *μ*.
5. $[x;t]\bar{\alpha }\mu$ if [*x*; *t*]*αμ* does not hold for *α* ∈ {∈_*γ*_, *q*_*δ*_, ∈_*γ*_∨*q*_*δ*_, ∈_*γ*_∧*q*_*δ*_}.

Note that, the case *α* = ∈_*γ*_∧*q*_*δ*_ is omitted. Because the set {[*x*; *t*] | [*x*; *t*]∈_*γ*_∧*q*_*δ*_} is empty for *μ*(*x*) ≤ *δ* that is, if *x* ∈ *S* and *r* ∈ (0,1] be such that [*x*; *t*]∈_*γ*_∧*q*_*δ*_ *μ*, then *μ*(*x*) ≥ *t* > *γ* and *μ*(*x*) + *t* > 2*δ*. It follows that 2(*x*) = (*x*) + (*x*) ≥ (*x*) + *t* > 2*δ* so that *μ*(*x*) > *δ* which is contradiction to *μ*(*x*) ≤ *δ*. This means that {[*x*; *t*] | [*x*; *t*]∈_*γ*_∧*q*_*δ*_} = *∅*.

**Definition 15**

A fuzzy subset *μ* of *S* is called an (*α*, *β*)*-fuzzy generalized bi-ideal* of *S* if it satisfies the following conditions:

(B1) (∀*x*, *y* ∈ *S*)  (∀*t* ∈ (*γ*, 1])([*y*; *t*]*αμ*⇒[*x*; *t*]*βμ* with *x* ≤ *y*).
(B2) (∀*x*, *a*, *y* ∈ *S*)  (∀*s*, *t* ∈ (*γ*, 1])  ([*x*; *s*]*αμ* and [*y*; *t*]*αμ*⇒[*xa* *y*; min⁡{*s*, *t*}]*βμ*).

**Example 16**

Consider the ordered semigroup *S* = {*a*, *b*, *c*, *d*, *e*} with the multiplication table and order relation as defined in Example 13. Define a fuzzy subset *μ* : *S* → [0,1] as follows:

(9) $$\begin{array}{c} \mu \left(x\right)=\{\begin{array}{cc} 0.80 & \text{if}\;x=a,\\ 0.70 & \text{if}\;x=b,\\ 0.60 & \text{if}\;x=c,\\ 0.40 & \text{if}\;x=d,\\ 0.30 & \text{if}\;x=e. \end{array} \end{array}$$

Then by Definition 15 *μ* is an (∈_0.30_, ∈_0.30_∨*q*_0.40_)-fuzzy generalized bi-ideal of *S*.

**Theorem 17**

A fuzzy subset *μ* of *S* is an (∈_*γ*_, ∈_*γ*_∨*q*_*δ*_)-fuzzy generalized bi-ideal of *S* if and only if the following conditions hold for all *x*, *a*, *y* ∈ *S*:

(B3) (*x* ≤ *y*)→(max⁡{*μ*(*x*), *γ*} ≥ min⁡{*μ*(*y*), *δ*}),
(B4) (max⁡{*μ*(*xa* *y*), *γ*} ≥ min⁡{*μ*(*x*), *μ*(*y*), *δ*}).

**Proof**

(B1)⇒(B3). If there exists *x*, *y* ∈ *S* with *x* ≤ *y* such that

(10) $$\begin{array}{c} \max\left\{\mu \left(x\right),\gamma \right\}<t\leq \min\left\{\mu \left(y\right),\delta \right\}, \end{array}$$

then *μ*(*y*) ≥ *t* > *γ*, *μ*(*x*) < *t*, and *μ*(*x*) + *t* < 2*t* ≤ 2*δ*; that is, [*y*; *t*]∈_*γ*_ *μ* but $[x;t]\bar{\in_{\gamma }\vee q_{\delta }}\mu$, a contradiction. Hence (B3) is valid.

(B3)⇒(B1). Assume that there exists *x*, *y* ∈ *S* with *x* ≤ *y* and *t* ∈ (*γ*, 1] such that [*y*; *t*]∈_*γ*_ *μ* but $\left[x;t\right]\bar{\in_{\gamma }\vee q_{\delta }}\mu$; then *μ*(*y*) ≥ *t* > *γ*, *μ*(*x*) < *t* and *μ*(*x*) + *t* < 2*δ*. It follows that *μ*(*x*) < *δ* and so max⁡{*μ*(*x*), *γ*} < min⁡{*t*, *δ*} ≤ min⁡{*μ*(*y*), *δ*}, a contradiction. Hence (B1) is valid.

(B2)⇒(B4). If there exists *x*, *a*, *y* ∈ *S* such that

(11) $$\begin{array}{c} \max\left\{\mu \left(xay\right),\gamma \right\}<r\leq \min\left\{\mu \left(x\right),\mu \left(y\right),\delta \right\}, \end{array}$$

then

(12) $$\begin{array}{c} \mu \left(x\right)\geq r>\gamma ,\;\;\mu \left(y\right)\geq r>\gamma ,\;\;\mu \left(xay\right)<r, \end{array}$$

and *μ*(*xa* *y*) + *r* < 2*r* ≤ 2*δ*; that is, [*x*; *r*]∈_*γ*_ *μ*, [*y*; *r*]∈_*γ*_ *μ* but $[xay;r]\bar{\in_{\gamma }\vee q_{\delta }}$, a contradiction. Hence max⁡{*μ*(*xa* *y*), *γ*} ≥ min⁡{*μ*(*x*), *μ*(*y*), *δ*} for all *x*, *y* ∈ *S*.

(B4)⇒(B2). Assume that there exist *x*, *a*, *y* ∈ *S* and *r*, *t* ∈ (*γ*, 1] such that [*x*; *r*]∈_*γ*_ *μ*, [*y*; *t*]∈_*γ*_ *μ* but $[xay;\min\{r,t\}]\bar{\in_{\gamma }\vee q_{\delta }}\mu$; then

(13) $$\begin{array}{c} \mu \left(x\right)\geq r>\gamma ,\;\;\mu \left(y\right)\geq t>\gamma ,\\ \mu \left(xay\right)<\min\left\{\mu \left(x\right),\mu \left(y\right),\delta \right\}, \end{array}$$

and *μ*(*xa* *y*) + min⁡{*r*, *t*} ≤ 2*δ*. It follows that *μ*(*xa* *y*) < *δ* and so

(14) $$\begin{array}{c} \max\left\{\mu \left(xay\right),\gamma \right\}<\min\left\{\mu \left(x\right),\mu \left(y\right),\delta \right\}, \end{array}$$

a contradiction. Hence (B2) is valid.

**Theorem 18**

The set $\mu_{\overset{-}{\gamma }}=\{x\in S|\mu (x)>\gamma \}$ is a generalized bi-ideal of *S*, whenever *μ* is an (*α*, ∈_*γ*_∨*q*_*δ*_)-fuzzy generalized bi-ideal of *S* (*α* ≠ ∈_*γ*_∧*q*_*δ*_) and 2*δ* = 1 + *γ*.

**Proof**

Assume that *μ* is an (*α*, ∈_*γ*_∨*q*_*δ*_)-fuzzy generalized bi-ideal of *S*. Let *a* ∈ *S*, $x,y\in \mu_{\overset{-}{\gamma }}$. Then *μ*(*x*) > *γ*, *μ*(*y*) > *γ*.

*Case  1.* Then [*x*; *μ*(*x*)]*αμ* and [*y*; *μ*(*y*)]*αμ*, where *α* ∈ {∈_*γ*_, ∈_*γ*_∨  *q*_*δ*_}. By (B2),

(15) $$\begin{array}{c} \left[xay;\min\left\{\mu \left(x\right),\mu \left(y\right)\right\}\right]\in_{\gamma }\vee q_{\delta }\mu . \end{array}$$

It follows that *μ*(*xa* *y*) ≥ min⁡{*μ*(*x*), *μ*(*y*)} > *γ* or *μ*(*xa* *y*) + {*μ*(*x*), *μ*(*y*)} > 2*δ*, and so *μ*(*xa* *y*)≥  min{*μ*(*x*), *μ*(*y*)} > *γ* or *μ*(*xa* *y*) > 2*δ* − min⁡{*μ*(*x*), *μ*(*y*)} ≥ 2*δ* − 1 = *γ*. Hence $xay\in \mu_{\overset{-}{\gamma }}$.

*Case  2.* Then [*x*; 1]*αμ* and [*y*; 1]*αμ*, where *α* = *q*_*δ*_, since 2*δ* = 1 + *γ*. Analogous to the proof of Case 1, we have $xay\in \mu_{\overset{-}{\gamma }}$. Similarly, for *x*, *y* ∈ *S* and *x* ≤ *y* if $y\in \mu_{\overset{-}{\gamma }}$, then $x\in \mu_{\overset{-}{\gamma }}$. Consequently, $\mu_{\overset{-}{\gamma }}$ is a generalized bi-ideal of *S*.

**Theorem 19**

Consider a fuzzy subset *μ* of *S* defined as follows:

(16) $$\begin{array}{c} \mu \left(x\right)=\{\begin{array}{ccc} \geq \delta , & if\;\;x\in A, & \\ \gamma , & if\;\;x\notin A, & \end{array} \end{array}$$

where *A* is a nonempty subset of *S*. Then  *A* is a generalized bi-ideal of *S* if and only if *μ* is an (*α*, ∈_*γ*_∨*q*_*δ*_)-fuzzy generalized bi-ideal of *S*.

**Proof**

Assume that *A* is a generalized bi-ideal of *S* and *x*, *y* ∈ *S* with *x* ≤ *y*, *t* ∈ (*γ*, 1] be such that [*y*; *t*]*αμ*. We consider the following cases.

*Case  1.* If [*y*; *t*]∈_*γ*_ *μ*, then *μ*(*y*) ≥ *t* > *γ*  follows that *y* ∈ *A*.

*Case  2.* If [*y*; *t*]*q*_*δ*_ *μ*, then *μ*(*y*) + *t* > 2*δ* and so *μ*(*y*) > 2*δ* − *t* ≥ 2*δ* − 1 = *γ*  follow that *y* ∈ *A*.

In the above two cases *y* ∈ *A* and hence *x* ∈ *A*. By definition of *μ* we have *μ*(*x*) ≥ *δ*. If *t* ≤ *δ*, then *μ*(*x*) ≥ *δ* ≥ *t* > *γ* and hence [*x*; *t*]∈_*γ*_ *μ* but if *t* > *δ*, then *μ*(*x*) + *t* ≥ *δ* + *t* > 2*δ*; that is, [*x*; *t*]*q*_*δ*_ *μ*. Thus for [*y*; *t*]∈_*γ*_ *μ* we have [*x*; *t*]∈_*γ*_∨*q*_*δ*_ *μ*.

Next we suppose that *x*, *y*, *a* ∈ *S* and *r*, *t* ∈ (*γ*, 1] be such that [*x*; *r*]*αμ* and [*y*; *t*]*αμ*. We consider the following four cases.

*Case  1.* If [*x*; *r*]∈_*γ*_ *μ* and [*y*; *t*]∈_*γ*_ *μ*, then *μ*(*x*) ≥ *r* > *γ* and *μ*(*y*) ≥ *t* > *γ* follow that *x*, *y* ∈ *A*.

*Case  2.* If [*x*; *r*]*q*_*δ*_ *μ* and [*y*; *t*]*q*_*δ*_ *μ*, then *μ*(*x*) + *r* > 2*δ* and *μ*(*y*) + *t* > 2*δ*  and so *μ*(*x*) > 2*δ* − *r* ≥ 2*δ* − 1 = *γ* and *μ*(*y*) > 2*δ* − *t* ≥ 2*δ* − 1 = *γ* follow that *x*, *y* ∈ *A*.

*Case  3.* Similarly, if [*x*; *r*]∈_*γ*_ *μ* and [*y*; *t*]*q*_*δ*_ *μ*, then *x*, *y* ∈ *A*.

*Case  4.* If [*x*; *r*]*q*_*δ*_ *μ* and [*y*; *t*]∈_*γ*_ *μ*, then *x*, *y* ∈ *A*.

Thus, in any case, *x*, *y* ∈ *A*. Hence *xa* *y* ∈ *A* and by definition we have that *μ*(*xa* *y*) ≥ *δ*. If min{*r*, *t*} ≤ *δ*, then *μ*(*xa* *y*) ≥ *δ* ≥ min⁡{*r*, *t*} > *γ*; that is,  [*xa* *y*; min⁡{*r*, *t*}]∈_*γ*_ *μ*. If min{*r*, *t*} > *δ*, then *μ*(*xa* *y*) + min⁡{*r*, *t*} > *δ* + *δ* = 2*δ*; that is, [*xa* *y*; min⁡{*r*, *t*}]*q*_*δ*_ *μ*. Therefore [*xa* *y*; min⁡{*r*, *t*}]∈_*γ*_∨*q*_*δ*_ *μ*.

Conversely, assume that *μ* is an (∈_*γ*_, ∈_*γ*_∨*q*_*δ*_)-fuzzy generalized bi-ideal of *S*. It is easy to see that $A=\mu_{\overset{-}{\gamma }}$. Hence, from Theorem 18 *A* is a generalized bi-ideal of *S*.

**Proposition 20**

Every (∈_*γ*_∨*q*_*δ*_, ∈_*γ*_∨*q*_*δ*_)-fuzzy generalized bi-ideal of *S* is an (∈_*γ*_, ∈_*γ*_∨*q*_*δ*_)-fuzzy generalized bi-ideal of *S*.

**Proof**

It is straightforward since [*x*; *r*]∈_*γ*_ *μ* implies [*x*; *r*]∈_*γ*_∨*q*_*δ*_ *μ* for all *x* ∈ *S* and *r* ∈ (*γ*, 1].

**Proposition 21**

Every (∈_*γ*_, ∈_*γ*_)-fuzzy generalized bi-ideal of *S* is an (∈_*γ*_, ∈_*γ*_∨*q*_*δ*_)-fuzzy generalized bi-ideal of *S*.

**Proof**

The proof is straightforward and is omitted here.

From the example given below we see that the converses of Propositions 20 and 21 may not be true in general.

**Example 22**

Consider the ordered semigroup *S* = {*a*, *b*, *c*, *d*, *e*} with the multiplication table and order relation as defined in Example 13. Define a fuzzy subset *μ* : *S* → [0,1] as follows:

(17) $$\begin{array}{c} \mu \left(x\right)=\{\begin{array}{cc} 0.90 & \text{if}\;x=a,\\ 0.70 & \text{if}\;x=b,\\ 0.40 & \text{if}\;x=d,\\ 0.60 & \text{if}\;x=c,\\ 0.30 & \text{if}\;x=e. \end{array} \end{array}$$

Then,

1. by Definition 15, *μ* is an (∈_0.30_, ∈_0.30_∨*q*_0.40_)-fuzzy generalized bi-ideal of *S*;
2. *μ* is not an (∈_0.30_, ∈_0.30_)-fuzzy generalized bi-ideal of *S*, since [*a*; 0.60]∈_0.30_ *μ* and [*b*; 0.50]∈_0.30_ *μ* but $\left[abb;\min\left\{0.60,0.50\right\}\right]=\left[d;0.50\right]\bar{\in_{0.30}}\mu$;
3. *μ* is not an (∈_0.30_∨*q*_0.60_, ∈_0.30_∨*q*_0.60_)-fuzzy generalized bi-ideal of *S*, since [*a*; 0.60]∈_0.30_∨*q*_0.60_ *μ* and [*b*; 0.5]∈_0.30_∨*q*_0.60_ *μ* but $[abb;\min\{0.60,0.50\}]=[d;0.50]\bar{\in_{0.30}\vee q_{0.60}}\mu$.

For any fuzzy subset *μ* of *S*, we define the following sets for all *t* ∈ (*γ*, 1]:

(18) $$\begin{array}{c} \mu_{t}=\left\{x\in S\;|\;\left[x;t\right]\in_{\gamma }\mu \right\},\\ \mu_{t}^{\delta }=\left\{x\in S\;|\;\left[x;t\right]q_{\delta }\mu \right\},\\ {\left[\mu \right]}_{t}^{\delta }=\left\{x\in S\;|\;\left[x;t\right]\in_{\gamma }\vee q_{\delta }\mu \right\}. \end{array}$$

It follows that [*μ*]_*t*_^*δ*^ = *μ*_*t*_ ∪ *μ*_*t*_^*δ*^.

The next theorem provides the relationship between (∈_*γ*_, ∈_*γ*_∨*q*_*δ*_)-fuzzy generalized bi-ideal and crisp generalized bi-ideal of *S*.

**Theorem 23**

For any fuzzy subset *μ*  of an ordered semigroup *S*, the following are equivalent:

1. *μ* is an (∈_*γ*_, ∈_*γ*_∨*q*_*δ*_)-fuzzy generalized bi-ideal of *S*,
2. *μ*_*t*_(≠*∅*) is a generalized bi-ideal of *S* for all *t* ∈ (*γ*, *δ*].

**Proof**

(1)⇒(2). Let *μ* be an (∈_*γ*_, ∈_*γ*_∨*q*_*δ*_)-fuzzy generalized bi-ideal of *S*. Let *x*, *y* ∈ *S* with *x* ≤ *y* and *t* ∈ (*γ*, *δ*] be such that *y* ∈ *μ*_*t*_. Then [*y*; *t*]∈_*γ*_ *μ* and since *μ* is an (∈_*γ*_, ∈_*γ*_∨*q*_*δ*_)-fuzzy generalized bi-ideal of *S*, therefore [*x*; *t*]∈_*γ*_∨*q*_*δ*_ *μ*. If [*x*; *t*]∈_*γ*_ *μ*, then *x* ∈ *μ*_*t*_ and if [*x*; *t*]*q*_*δ*_ *μ*, then *μ*(*x*) > 2*δ* − *t* > *t* > *γ*; that is,  *x* ∈ *μ*_*t*_.

Let *x*, *y*, *a* ∈ *S* be such that *x*, *y* ∈ *μ*_*t*_ for some *t* ∈ (*γ*, *δ*]. Then [*x*; *t*]∈_*γ*_ *μ* and [*y*; *t*]∈_*γ*_ *μ*, and since *μ* is an (∈_*γ*_, ∈_*γ*_∨*q*_*δ*_)-fuzzy generalized bi-ideal of *S*, therefore [*xa* *y*; *t*]∈_*γ*_∨*q*_*δ*_ *μ*. If [*xa* *y*; *t*]∈_*γ*_ *μ*, then *xa* *y* ∈ *μ*_*t*_ and if [*xa* *y*; *t*]*q*_*δ*_ *μ*, then *μ*(*xa* *y*) > 2*δ* − *t* > *t* > *γ*; that is,  *xa* *y* ∈ *μ*_*t*_. Therefore *μ*_*t*_ is a generalized bi-ideal of *S*.

(2)⇒(1). Assume that *μ*_*t*_(≠*∅*) is a generalized bi-ideal of *S* for all *t* ∈ (*γ*, *δ*]. Let *x*, *y* ∈ *S* with *x* ≤ *y* and max⁡{*μ*(*x*), *γ*} < min⁡{*μ*(*y*), *δ*}; then there exists *t* ∈ (*γ*, *δ*] such that max⁡{*μ*(*x*), *γ*} < *t* ≤ min⁡{*μ*(*y*), *δ*}; this follows that [*y*; *t*]∈_*γ*_ *μ*; that is, *y* ∈ *μ*_*t*_ but $x\bar{\in }\mu_{t}$, a contradiction. Therefore, max⁡{*μ*(*x*), *γ*} ≥ min⁡{*μ*(*y*), *δ*} for all *x*, *y* ∈ *S* with *x* ≤ *y*. Let *x*, *y*, *a* ∈ *S* and max⁡{*μ*(*xa* *y*), *γ*} < min⁡{*μ*(*x*), *μ*(*y*), *δ*}; then max⁡{*μ*(*xa* *y*), *γ*} < *t* ≤ min⁡{*μ*(*x*), *μ*(*y*), *δ*} for some *t* ∈ (*γ*, *δ*]. This implies that *x* ∈ *μ*_*t*_ and *y* ∈ *μ*_*t*_ but $xay\bar{\in }\mu_{t}$, a contradiction. Therefore,

(19) $$\begin{array}{c} \max\left\{\mu \left(xay\right),\gamma \right\}\geq \min\left\{\mu \left(x\right),\mu \left(y\right),\delta \right\}. \end{array}$$

Consequently, *μ* is an (∈_*γ*_, ∈_*γ*_∨*q*_*δ*_)-fuzzy generalized bi-ideal.

**Theorem 24**

For any fuzzy subset *μ*  of an ordered semigroup *S*, then *μ* is an (∈_*γ*_, ∈_*γ*_∨*q*_*δ*_)-fuzzy generalized bi-ideal of *S* if and only if *μ*_*r*_^*δ*^(≠*∅*) is a generalized bi-ideal of *S* for all *t* ∈ (*δ*, 1].

**Proof**

Let *μ* be an (∈_*γ*_, ∈_*γ*_∨*q*_*δ*_)-fuzzy generalized bi-ideal of *S*. Let *x*, *y* ∈ *S* with *x* ≤ *y* and *t* ∈ (*δ*, 1] be such that *y* ∈ *μ*_*t*_^*δ*^. Then [*y*; *t*]*q*_*δ*_ *μ*; that is,  *μ*(*y*) > 2*δ* − *t*. Since *t* ∈ (*δ*, 1], we have 2*δ* − *t* < *δ* < *t* and *μ* is an (∈_*γ*_, ∈_*γ*_∨*q*_*δ*_)-fuzzy generalized bi-ideal of *S*; therefore

(20) max⁡{μ(x),γ}≥min⁡{μ(y),δ}≥min⁡{2δ−t,δ}=2δ−t;

that is, *μ*(*x*) ≥ 2*δ* − *t*. Hence *x* ∈ *μ*_*t*_^*δ*^.

Let *x*, *y*, *a* ∈ *S* be such that *x*, *y* ∈ *μ*_*t*_^*δ*^ for some *t* ∈ (*δ*, 1]. Then [*x*; *t*]*q*_*δ*_ *μ*, [*y*; *t*]*q*_*δ*_ *μ*; that is, *μ*(*x*) > 2*δ* − *t*, that is, *μ*(*y*) > 2*δ* − *t*. Since *μ* is an (∈_*γ*_, ∈_*γ*_∨*q*_*δ*_)-fuzzy generalized bi-ideal of *S*, therefore

(21) max⁡{μ(xay),γ}≥min⁡{μ(x),μ(y),δ}≥min⁡{2δ−t,2δ−t,δ}=2δ−t;

that is, *μ*(*xa* *y*) ≥ 2*δ* − *t*. Hence *xa* *y* ∈ *μ*_*t*_^*δ*^. Consequently, *μ*_*t*_^*δ*^ is generalized bi-ideal.

Conversely, let *μ*_*t*_^*δ*^(≠*∅*) be a generalized bi-ideal of *S* for all *t* ∈ (*δ*, 1]. Let *x*, *y* ∈ *S* with *x* ≤ *y* and max⁡{*μ*(*x*), *γ*} < *t* = min⁡{*μ*(*y*), *δ*}; this follows that *y* ∈ *μ*_*t*_^*δ*^ but $x\bar{\in }\mu_{t}^{\delta }$, a contradiction. Therefore, max⁡{*μ*(*x*), *γ*} ≥ min⁡{*μ*(*y*), *δ*} for all *x*, *y* ∈ *S* with *x* ≤ *y*. Let *x*, *y*, *a* ∈ *S* and max⁡{*μ*(*xa* *y*), *γ*} < *t* = min⁡{*μ*(*x*), *μ*(*y*), *δ*}; this implies that *x* ∈ *μ*_*t*_^*δ*^ and *y* ∈ *μ*_*t*_^*δ*^ but $xay\bar{\in }\mu_{t}^{\delta }$, a contradiction. Therefore, max⁡{*μ*(*xa* *y*), *γ*} ≥ min⁡{*μ*(*x*), *μ*(*y*), *δ*}. Consequently, *μ* is an (∈_*γ*_, ∈_*γ*_∨*q*_*δ*_)-fuzzy generalized bi-ideal.

**Theorem 25**

A fuzzy subset *μ*  of an ordered semigroup *S* is an (∈_*γ*_, ∈_*γ*_∨*q*_*δ*_)-fuzzy generalized bi-ideal of *S* if and only if [*μ*]_*t*_^*δ*^(≠*∅*) is a generalized bi-ideal of *S* for all *t* ∈ (*γ*, 1].

**Proof**

The proof follows from Theorems 23 and 24.

**Definition 26**

A fuzzy subset *μ* of *S* is called an (*α*, *β*)*-fuzzy subsemigroup* if it satisfies the following condition:

(B5) (∀*x*, *y* ∈ *S*)  (∀*s*, *t* ∈ (*γ*, 1])  ([*x*; *s*]*αμ* and [*y*; *t*]*αμ*⇒[*xy*; min⁡{*s*, *t*}]*βμ*).

**Definition 27**

A fuzzy subset *μ* of *S* is called an (*α*, *β*)-*fuzzy bi-ideal* of *S* if it is (*α*, *β*)-fuzzy subsemigroup and (*α*, *β*)-fuzzy generalized bi-ideal of *S*.

**Theorem 28**

A fuzzy subset *μ* of *S* is an (∈_*γ*_, ∈_*γ*_∨*q*_*δ*_)-fuzzy bi-ideal of *S* if and only if the following conditions hold for all *x*, *a*, *y* ∈ *S*:

(B6) (*x* ≤ *y*)→(max⁡{*μ*(*x*), *γ*} ≥ min⁡{*μ*(*y*), *δ*}),
(B7) (max⁡{*μ*(*xy*), *γ*} ≥ min⁡{*μ*(*x*), *μ*(*y*), *δ*}),
(B8) (max⁡{*μ*(*xa* *y*), *γ*} ≥ min⁡{*μ*(*x*), *μ*(*y*), *δ*}).

**Proof**

(B5)⇒(B7). If there exists *x*, *y* ∈ *S* such that

(22) $$\begin{array}{c} \max\left\{\mu \left(xy\right),\gamma \right\}<r\leq \min\left\{\mu \left(x\right),\mu \left(y\right),\delta \right\}, \end{array}$$

then

(23) $$\begin{array}{c} \mu \left(x\right)\geq r>\gamma ,\;\;\mu \left(y\right)\geq r>\gamma ,\;\;\mu \left(xy\right)<r, \end{array}$$

and *μ*(*xy*) + *r* < 2*r* ≤ 2*δ*; that is, [*x*; *r*]∈_*γ*_ *μ*, [*y*; *r*]∈_*γ*_ *μ* but $[xy;r]\bar{\in_{\gamma }\vee q_{\delta }}$, a contradiction. Hence max⁡{*μ*(*xy*), *γ*} ≥ min⁡{*μ*(*x*), *μ*(*y*), *δ*} for all *x*, *y* ∈ *S*.

(B7)⇒(B5). Assume that there exist *x*, *y* ∈ *S* and *r*, *t* ∈ (*γ*, 1] such that [*x*; *r*]∈_*γ*_ *μ*, [*y*; *t*]∈_*γ*_ *μ* but $[xy;\min\{r,t\}]\bar{\in_{\gamma }\vee q_{\delta }}\mu$; then

(24) μ(x)≥r>γ,  μ(y)≥t>γ,μ(xy)<min⁡{μ(x),μ(y),δ},

and *μ*(*xy*) + min⁡{*r*, *t*} ≤ 2*δ*. It follows that *μ*(*xy*) < *δ* and so

(25) $$\begin{array}{c} \max\left\{\mu \left(xy\right),\gamma \right\}<\min\left\{\mu \left(x\right),\mu \left(y\right),\delta \right\}, \end{array}$$

a contradiction. Hence (B5) is valid.

The remaining proof follows from Theorem 17.

Next, the relationship between (∈_*γ*_, ∈_*γ*_∨*q*_*δ*_)-fuzzy bi-ideals and (∈_*γ*_, ∈_*γ*_∨*q*_*δ*_)-fuzzy generalized bi-ideals is provided.

**Corollary 29**

Every (∈_*γ*_, ∈_*γ*_∨*q*_*δ*_)-fuzzy bi-ideal *μ* of *S* is (∈_*γ*_, ∈_*γ*_∨*q*_*δ*_)-fuzzy generalized bi-ideal.

**Proof**

The proof is straightforward and is omitted.

The converse of the Corollary 29 is not true in general as shown in the following example.

**Example 30**

Consider the ordered semigroup *S* = {0, *x*, *y*, *z*} with the multiplication table and order relation as shown in Example 3. Define a fuzzy subset *μ* of *S* as follows:

(26) $$\begin{array}{c} \mu \left(a\right)=\{\begin{array}{cc} 0.90 & \text{if}\;a=0,\\ 0.70 & \text{if}\;a=y,\\ 0.25 & \text{if}\;a=x,z. \end{array} \end{array}$$

Then by Definition 15 *μ* is an (∈_0.28_, ∈_0.28_∨*q*_0.30_)*-*fuzzy generalized bi-ideal of *S* but not an (∈_0.28_, ∈_0.28_∨*q*_0.30_)-fuzzy bi-ideal, since [*y*; 0.28]∈_0.28_ *μ* but $\left[yy;\min\{0.28,0.28\}\right]=[x;0.28]\bar{\in_{0.28}\vee q_{0.30}}\mu$.

**Theorem 31**

A nonempty subset *A* of an ordered semigroup *S* is a generalized bi-ideal of *S* if and only if

(27) $$\begin{array}{c} \chi_{A}:S⟶\left[0,1\right]\;|\;x⟼\chi_{A}\left(x\right)=\{\begin{array}{cc} 1 & if\;x\in A,\\ 0 & if\;x\notin A. \end{array} \end{array}$$

is an (∈_*γ*_, ∈_*γ*_∨*q*_*δ*_)-fuzzy generalized bi-ideal of *S*.

**Proof**

The proof is straightforward and is omitted.

## 4. ($\bar{\beta },\;\bar{\alpha }$)-Fuzzy Bi-Ideals

In the last couple of decades, the importance of fuzzification of ordered semigroups and related structures is increased due to the pioneering role of aforementioned structures in advanced fields like computer science, error correcting codes, and fuzzy automata. In contribution to this fact, we define and investigate ($\bar{\beta },\bar{\alpha }$)-fuzzy generalized bi-ideals of ordered semigroups, where $\bar{\alpha }$, $\bar{\beta }\in \{\bar{\in_{\gamma }},\bar{q_{\delta }},\bar{\in_{\gamma }}\wedge \bar{q_{\delta }},\bar{\in_{\gamma }}\vee \bar{q_{\delta }}\}$ with $\bar{\beta }\neq \bar{\in_{\gamma }}\wedge \bar{q_{\delta }}$ and discussed some important results of ordered semigroups in terms of ($\bar{\beta },\bar{\alpha }$)-fuzzy generalized bi-ideals.

**Definition 32**

A fuzzy subset *μ* of *S* is called $\left(\bar{\beta },\bar{\alpha }\right)$-fuzzy generalized bi-ideal of *S* if it satisfies the following conditions:

(B9) $(\forall x,y\in S)\;\;(\forall r\in (\gamma ,1])\;\;([x;r]\bar{\beta }\mu \Rightarrow [y;r]\bar{\alpha }\mu$ with *x* ≤ *y*),
(B10) $(\forall x,a,y\in S)\;\;(\forall r,t\in (\gamma ,1])\;\;([xay;\min\{r,t\}]\bar{\beta }\mu \Rightarrow [x;r]\bar{\alpha }\mu$ or $\left[y;t]\bar{\alpha }\mu \right)$.

The case when $\bar{\beta }=\bar{\in_{\gamma }}\wedge \bar{q_{\delta }}$ can be omitted since for a fuzzy subset *μ* of *S* such that *μ*(*x*) ≥ *δ* for any *x* ∈ *S* in the case $[x;r]\bar{\in_{\gamma }}\wedge \bar{q_{\delta }}\mu$ we have *μ*(*x*) < *r* and *μ*(*x*) + *r* < 2*δ*. Thus *μ*(*x*) + *μ*(*x*) < *μ*(*x*) + *r* ≤ 2*δ*, which implies *μ*(*x*) < *δ*. This means that $\{[x;r]:[x;r]\bar{\in_{\gamma }}\wedge \bar{q_{\delta }}\mu \}=\emptyset$.

**Example 33**

Consider an ordered semigroup *S* = {*a*, *b*, *c*, *d*, *e*} with the following multiplication table and order relation:

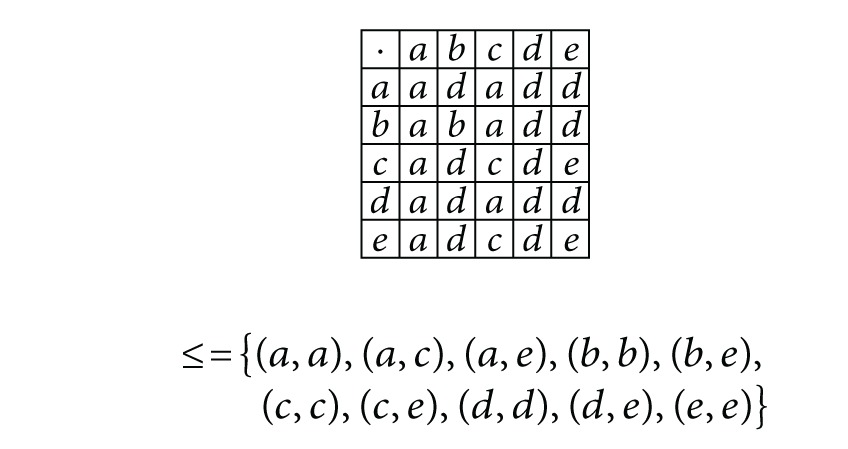
(28)

Define a fuzzy subset *μ* : *S* → [0,1] as follows:

(29) $$\begin{array}{c} \mu \left(x\right)=\{\begin{array}{cc} 0.60 & \text{if}\;x=a,\\ 0.50 & \text{if}\;x=b,\\ 0.70 & \text{if}\;x=c,\\ 0.80 & \text{if}\;x=d,\\ 0.90 & \text{if}\;x=e. \end{array} \end{array}$$

Then by Definition 32 *μ* is an ($\bar{\in_{0.3}},\bar{\in_{0.3}}\vee \bar{q_{0.6}}$)-fuzzy generalized bi-ideal of *S*.

**Theorem 34**

A fuzzy subset *μ* of *S* is an ($\bar{\in_{\gamma }},\bar{\in_{\gamma }}\vee \bar{q_{\delta }}$)-fuzzy generalized bi-ideal of *S* if and only if the following conditions hold:

(B11) (∀*x*, *y* ∈ *S*)  (max⁡{*μ*(*x*), *δ*} ≥ *μ*(*y*) with *x* ≤ *y*),
(B12) (∀*x*, *a*, *y* ∈ *S*)  (max⁡{*μ*(*xa* *y*), *δ*} ≥ min⁡{*μ*(*x*), *μ*(*y*)}).

**Proof**

(B9)⇒(B11). Assume that there exists *x*, *y* ∈ *S* with *x* ≤ *y* such that max⁡{*μ*(*x*), *δ*} < *μ*(*y*). Then max⁡{*μ*(*x*), *δ*} < *t* ≤ *μ*(*y*) for some *t* ∈ (*γ*, 1] follows that $[x;r]\bar{\in_{\gamma }}\mu$ but $[y;r]\bar{\bar{\in_{\gamma }}\vee \;\bar{q_{\delta }}}\mu$, a contradiction. Hence max⁡{*μ*(*x*), *δ*} ≥ *μ*(*y*) for all *x*, *y* ∈ *S* with *x* ≤ *y*.

(B11)⇒(B9). Assume that there exists *x*, *y* ∈ *S* with *x* ≤ *y* and *t* ∈ (*γ*, 1] such that $[x;r]\bar{\in_{\gamma }}\mu$ but $\left[y;r\right]\bar{\bar{\in_{\gamma }}\vee \bar{q_{\delta }}}\mu$; then *μ*(*x*) < *t*, *μ*(*y*) ≥ *t*, and *μ*(*y*) + *t* > 2*δ* and hence *μ*(*y*) > *δ*. This follows that

(30) $$\begin{array}{c} \mu \left(y\right)\geq \max\left\{t,\delta \right\}>\max\left\{\mu \left(x\right),\delta \right\}, \end{array}$$

a contradiction. Hence $[x;r]\bar{\in_{\gamma }}\mu$ implies $[y;r]\bar{\in_{\gamma }}\vee \bar{q_{\delta }}\mu$.

(B10)⇒(B12). If *x*, *a*, *y* ∈ *S* such that max⁡{*μ*(*xa* *y*), *δ*} < min⁡{*μ*(*x*), *μ*(*y*)}, then there exists *t* ∈ (*γ*, 1] such that max⁡{*μ*(*xa* *y*), *δ*} < *t* ≤ min⁡{*μ*(*x*), *μ*(*y*)}. It follows that $[xay;r]\bar{\in_{\gamma }}\mu$ but $[x;r]\bar{\bar{\in_{\gamma }}\vee \bar{q_{\delta }}}\mu$ and $[y;r]\bar{\bar{\in_{\gamma }}\vee \bar{q_{\delta }}}\mu$, a contradiction. Hence max⁡{*μ*(*xa* *y*), *δ*} ≥ min⁡{*μ*(*x*), *μ*(*y*)} for all *x*, *a*, *y* ∈ *S*.

(B12)⇒(B10). Assume that there exist *x*, *a*, *y* ∈ *S* and *r*, *t* ∈ (*γ*, 1] such that $[xay;\min\{r,t\}]\bar{\in_{\gamma }}\mu$ but $[x;r]\bar{\bar{\in_{\gamma }}\vee \bar{q_{\delta }}}\mu$ and $\left[y;r\right]\bar{\bar{\in_{\gamma }}\vee \bar{q_{\delta }}}\mu$; then *μ*(*xa* *y*) < min⁡{*r*, *t*}, *μ*(*x*) ≥ *r*, *μ*(*y*) ≥ *t*, *μ*(*x*) + *r* > 2*δ*, and *μ*(*y*) + *r* > 2*δ*. It follows that *μ*(*x*) > *δ* and *μ*(*y*) > *δ*, and so

(31) min⁡{μ(x),μ(y)}≥max⁡{min⁡{r,t},δ}>max⁡{μ(xay),δ},

a contradiction. Hence (B10) is valid.

**Definition 35**

A fuzzy subset *μ* of *S* is called $\left(\bar{\beta },\bar{\alpha }\right)$-fuzzy subsemigroup of *S* if it satisfies the following conditions:

(B13) $(\forall x,y\in S)\;\;(\forall r,t\in (\gamma ,1])([xy;\min\{r,t\}]\bar{\beta }\mu \Rightarrow [x;r]\bar{\alpha }\mu$ or $\left[y;t]\bar{\alpha }\mu \right)$.

**Definition 36**

A fuzzy subset *μ* of *S* is called $\left(\bar{\beta },\bar{\alpha }\right)$-fuzzy bi-ideal of *S* if it is $\left(\bar{\beta },\bar{\alpha }\right)$-fuzzy subsemigroup and $\left(\bar{\beta },\bar{\alpha }\right)$-fuzzy generalized bi-ideal of *S*.

**Theorem 37**

A fuzzy subset *μ* of *S* is an ($\bar{\in_{\gamma }},\bar{\in_{\gamma }}\vee \bar{q_{\delta }}$)-fuzzy generalized bi-ideal of *S* if and only if the following conditions hold:

(B14) (∀*x*, *y* ∈ *S*)  (max⁡{*μ*(*x*), *δ*} ≥ *μ*(*y*) with *x* ≤ *y*),
(B15) (∀*x*, *y* ∈ *S*)  (max⁡{*μ*(*xy*), *δ*} ≥ min⁡{*μ*(*x*), *μ*(*y*)})
(B16) (∀*x*, *a*, *y* ∈ *S*)  (max⁡{*μ*(*xa* *y*), *δ*} ≥ min⁡{*μ*(*x*), *μ*(*y*)}).

**Proof**

(B13)⇒(B15). Suppose *x*, *y* ∈ *S* such that

(32) $$\begin{array}{c} \max\left\{\mu \left(xy\right),\delta \right\}<\min\left\{\mu \left(x\right),\mu \left(y\right)\right\}. \end{array}$$

Then there exists *t* ∈ (*γ*, 1] such that max⁡{*μ*(*xy*), *δ*} < *t* ≤ min⁡{*μ*(*x*), *μ*(*y*)}. It follows that $[xy;r]\bar{\in_{\gamma }}\mu$ but $[x;r]\bar{\bar{\in_{\gamma }}\vee \bar{q_{\delta }}}\mu$ and $[y;r]\bar{\bar{\in_{\gamma }}\vee \bar{q_{\delta }}}\mu$, a contradiction. Hence max⁡{*μ*(*xy*), *δ*} ≥ min⁡{*μ*(*x*), *μ*(*y*)} for all *x*, *y* ∈ *S*.

(B15)⇒(B13). If there exist *x*, *y* ∈ *S* and *r*, *t* ∈ (*γ*, 1] such that $[xy;\min\{r,t\}]\bar{\in_{\gamma }}\mu$ but $[x;r]\bar{\bar{\in_{\gamma }}\vee \bar{q_{\delta }}}\mu$ and $[y;r]\bar{\bar{\in_{\gamma }}\vee \bar{q_{\delta }}}\mu$, then *μ*(*xy*) < min⁡{*r*, *t*}, *μ*(*x*) ≥ *r*, *μ*(*y*) ≥ *t*, *μ*(*x*) + *r* > 2*δ*, and *μ*(*y*) + *r* > 2*δ*. It follows that *μ*(*x*) > *δ* and *μ*(*y*) > *δ*, and so

(33) $$\begin{array}{c} \min\left\{\mu \left(x\right),\mu \left(y\right)\right\}\geq \max\left\{\min\left\{r,t\right\},\delta \right\}>\max\left\{\mu \left(xy\right),\delta \right\}, \end{array}$$

a contradiction. Hence (B13) is valid. The remaining proof follows from Theorem 34.

**Corollary 38**

Every ($\bar{\in_{\gamma }},\bar{\in_{\gamma }}\vee \bar{q_{\delta }}$)-fuzzy bi-ideal *μ* of *S* is ($\bar{\in_{\gamma }},\bar{\in_{\gamma }}\vee \bar{q_{\delta }}$)-fuzzy generalized bi-ideal.

**Proof**

The proof is straightforward and is omitted here.

**Theorem 39**

The set $\mu_{\bar{\delta }}=\{x\in S|\mu (x)>\delta \}$ is a generalized bi-ideal of *S* whenever *μ* is an $\left(\bar{\beta },\bar{\in_{\gamma }}\vee \bar{q_{\delta }}\right)$-fuzzy generalized bi-ideal of *S*.

**Proof**

Assume that *μ* is an $\left(\bar{\beta },\bar{\in_{\gamma }}\vee \bar{q_{\delta }}\right)$-fuzzy generalized bi-ideal of *S*. Let *x*, *y* ∈ *S* with *x* ≤ *y* be such that $y\in \mu_{\bar{\delta }}$; then *μ*(*y*) > *δ*. As *μ* is an $\left(\bar{\beta },\bar{\in_{\gamma }}\vee \bar{q_{\delta }}\right)$-fuzzy generalized bi-ideal of *S*, therefore

(34) max⁡{μ(x),δ}≥μ(y)>δ;

that is, *μ*(*x*) > *δ* and hence $x\in \mu_{\bar{\delta }}$.

Let *x*, *a*, *y* ∈ *S* be such that $x,y\in \mu_{\bar{\delta }}$; then *μ*(*x*) > *δ*, *μ*(*y*) > *δ*, and by (B8)

(35) max⁡{μ(xay),δ}≥min⁡{μ(x),μ(y)}>min⁡{δ,δ};

that is, $xay\in \mu_{\bar{\delta }}$. Therefore, $\mu_{\bar{\delta }}$ is a generalized bi-ideal of *S*.

**Theorem 40**

Consider a fuzzy subset *μ* of *S* defined as

(36) $$\begin{array}{c} \mu \left(x\right)=\{\begin{array}{cc} 1, & if\;x\in A,\\ \delta , & if\;x\notin A. \end{array} \end{array}$$

Then *A* is a generalized bi-ideal of *S* if and only if *μ* is an $\left(\bar{\beta },\bar{\in_{\gamma }}\vee \bar{q_{\delta }}\right)$-fuzzy generalized bi-ideal of *S*.

**Proof**

Assume that *A* is a generalized bi-ideal of *S*. Let *x*, *a*, *y* ∈ *S* and *r*, *t* ∈ (*δ*, 1] be such that $[xay;\min\{r,t\}]\bar{\beta }\mu$. Then we have the following three cases.

*Case  1.* $[xay;\min\{r,t\}]\bar{\in_{\gamma }}\mu$. Then *μ*(*xa* *y*) < min⁡{*r*, *t*} ≤ 1 and so *μ*(*xa* *y*) = *δ* < min⁡{*r*, *t*}; that is, *xa* *y* ∉ *A*. It follows that *x* ∉ *A* or *y* ∉ *A*, and so *μ*(*x*) = *δ* < *r* or *μ*(*y*) = *δ* < *t*. Hence $[x;r]\bar{\in_{\gamma }}\mu$ or $\left[y;t\right]\bar{\in_{\gamma }}\mu$; that is, $[x;r]\bar{\in_{\gamma }}\vee \bar{q_{\delta }}\mu$ or $[y;t]\bar{\in_{\gamma }}\vee \bar{q_{\delta }}\mu$.

*Case  2.* $[xay;\min\{r,t\}]\bar{q_{\delta }}\mu$. Then *μ*(*xa* *y*) + min⁡{*r*, *t*} ≤ 2*δ*. If *μ*(*xa* *y*) = *δ*, analogous to the proof of Case 1, we have $[x;r]\bar{\in_{\gamma }}\vee \bar{q_{\delta }}\mu$ or $[y;t]\bar{\in_{\gamma }}\vee \bar{q_{\delta }}\mu$. If *μ*(*xa* *y*) = 1; then max⁡{*μ*(*x*), *μ*(*y*)} + min⁡{*r*, *t*}≤ 1 + min⁡{*r*, *t*} =  *μ*(*xa* *y*) + min⁡{*r*, *t*} ≤ 2*δ*. It follows that *μ*(*x*) + *r* ≤ 2*δ* or *μ*(*y*) + *t* ≤ 2*δ*. Hence $[x;r]\bar{q_{\delta }}\mu$ or $\left[y;t\right]\bar{q_{\delta }}\mu$; that is, $[x;r]\bar{\in_{\gamma }}\vee \bar{q_{\delta }}\mu$ or $[y;t]\bar{\in_{\gamma }}\vee \bar{q_{\delta }}\mu$.

*Case  3.* $[xay;\min\{r,t\}]\bar{\in_{\gamma }}\vee \bar{q_{\delta }}\mu$. Then $[xay;\min\{r,t\}]\bar{\in_{\gamma }}\mu$ or $[xay;\min\{r,t\}]\bar{q_{\delta }}\mu$. Hence $[x;r]\bar{\in_{\gamma }}\vee \bar{q_{\delta }}\mu$ or $[y;t]\bar{\in_{\gamma }}\vee \bar{q_{\delta }}\mu$ as in Cases  1 and  2.

In a similar way we can show that $[x;r]\bar{\beta }\mu$ implies that $[y;r]\bar{\in_{\gamma }}\vee \bar{q_{\delta }}\mu$ for all *x*, *y* ∈ *S* with *x* ≤ *y*.

Conversely, assume that *μ* is a ($\bar{\in_{\gamma }},\bar{\in_{\gamma }}\vee \bar{q_{\delta }}$)-fuzzy generalized bi-ideal of *S*. It is easy to see that $A=\mu_{\bar{\delta }}$. Hence by Theorem 39 *A* is a generalized bi-ideal of *S*.

## 5. Conclusion

Due to the significant role of ordered semigroups and their different characterizations in several applied fields such as control engineering, fuzzy automata, coding theory, and computer science, the latest research has been carried out in the last few decades by considering various characterizations of ordered semigroups in terms of different types of fuzzy ideals. In this paper, we determined a more generalized form of Davvaz and Khan [9] approach of fuzzy generalized bi-ideals and introduced (∈_*γ*_, ∈_*γ*_∨*q*_*δ*_)-fuzzy generalized bi-ideals and ($\bar{\in_{\gamma }},\bar{\in_{\gamma }}\vee \bar{q_{\delta }}$)-fuzzy generalized bi-ideals. Further, several characterization theorems of ordered semigroups in terms of these notions are provided. The relationship between ordinary generalized bi-ideals and fuzzy generalized bi-ideals of type (∈_*γ*_, ∈_*γ*_∨*q*_*δ*_) is also constructed.
